# Description of *Oculogryphus shuensis* sp. n. (Coleoptera, Lampyridae), the first species of the genus in the Sino-Japanese realm, with a modified key to the subfamily Ototretinae

**DOI:** 10.3897/zookeys.378.6435

**Published:** 2014-02-06

**Authors:** Ming-Luen Jeng, Michael S. Engel

**Affiliations:** 1 Department of Biology, National Museum of Natural Science, No. 1, Guanqian Rd., Taichung City 40453, Taiwan, R.O.C.; 2 Division of Entomology, Natural History Museum, and Department of Ecology & Evolutionary Biology, University of Kansas, 1501 Crestline Drive – Suite 140, Lawrence, Kansas 66045, USA

**Keywords:** *Oculogryphus*, Lampyridae, Ototretinae, *Stenocladius*, China, key

## Abstract

A new species of the lampyrid genus *Oculogryphus* Jeng, Engel, and Yang, *O. shuensis* **sp. n.** from China (Sichuan Province) is described and figured. The genus previously was known only from Vietnam, and the new species is the first representative of the genus in the Sino-Japanese zoogeographic realm. Some morphological variations of *Oculogryphus* and the allied genus *Stenocladius* are discussed and a modification to the most recent key to ototretine genera is proposed to accommodate *Oculogryphus*.

## Introduction

*Oculogryphus* is a small beetle genus currently composed of two species known only from northern Vietnam (Jeng et al. 2007, 2011). The genus is morphologically distinctive in having enlarged compound eyes which are closely approximate ventrally and deeply emarginate on the posterior upper margin laterally. It was thought to be en enigmatic taxon with a mosaic of features intermingling those of Rhagophthalmidae, Luciolinae, Lampyrinae, and the ototretine-ototretadriline complex when established (Jeng et al. 2007). Subsequently the genus was revealed to be closely related to the ototretine genus *Stenocladius* Fairmaire s.str. based on a comprehensive phylogenetic study of Lampyridae based on male morphology (Jeng 2008, Jeng et al. 2011). In the most current revision of Ototretinae, Janisova and Bocakova (2013) synonymized the subfamily Ototretadrilinae and accordingly redefined the limits of the group. Eighteen ototretine genera were reviewed or revised, diagnostically characterized, and a key based on male characters, especially of aedeagal morphology, was given, but *Oculogryphus* was overlooked.

Here we describe a third species of the genus, recently collected in Sichuan Province, China. The new species is the first representative of the genus in the Sino-Japanese zoogeographic realm (*sensu* Holt et al. 2013). Some morphological variations of *Oculogryphus* and *Stenocladius* are discussed in detail and a modification to Janisova and Bocakova’s (2013) key to ototretine genera is proposed so as to include *Oculogryphus*.

## Material and methods

The methodology and morphological terminology used herein follows that of Jeng et al. (2007, 2011). The body length (BL) is the sum of the pronotal and elytral lengths (PL and EL, respectively) plus length of those exposed portions of the head from the pronotum. Body width is considered as twice the elytral width (BW = 2EW). Pronotal width is abbreviated as PW. The nomenclature of the hind wing venation follows that of Kukalová-Peck and Lawrence (2004). In reporting label data the symbol “/” indicates separate lines on a single label. The holotype will be deposited in the insect collection of the Chinese Academy of Sciences, Beijing (CAS) and the paratype in the National Museum of Natural Science (NMNS), Taichung, Taiwan.

## Results

### Oculogryphus shuensis sp. n.

http://zoobank.org/275FCCE6-4581-427C-9717-C2285D2AD8BD

http://species-id.net/wiki/Oculogryphus_shuensis

Figs 1 – 5

#### Holotype.

♂, “CHINA: Sichuan Province/ Chongqing City, Jijiang Distr./ Shuikousi, by net/ 24.VI.2013/ YT Wang leg.

#### Paratype.

1 ♂, “CHINA: Sichuan Province/ Chongqing City, Jiangjin Distr./ Dawopu, by FIT/, 22.VI.2013/ YT Wang leg.

#### Type-locality.

China, Sichuan Province, Chongqing City, Jijiang Distr., Shuikousi, 18°22'N, 106°13'E.

#### Diagnosis.

**Figures 1–2.**
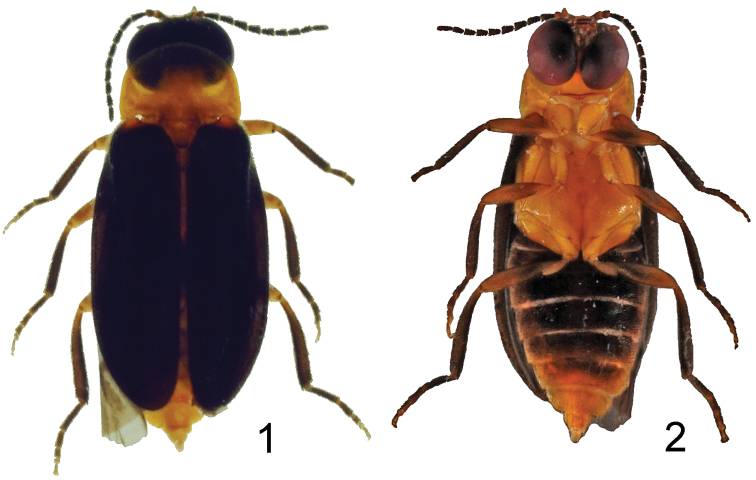
Habitus of holotype of *Oculogryphus shuensis* sp. n. **1** dorsal aspect **2** ventral aspect.

In comparison with the other two documented species, *Oculogryphus shuensis* sp. n. more closely resembles *Oculogryphus bicolor* Jeng, Engel et Branham than it does *Oculogryphus fulvus* Jeng. For example, the new species has a broader elytral epipleura, more slender metatibia, and more elongate parameres, much like *Oculogryphus bicolor*. The new species can be differentiated easily from the others by its highly contrasting bicoloration on the dorsum (Fig. 1) and black abdominal ventrites 1–5 (Fig. 2). It additionally differs from *Oculogryphus bicolor* by having a subparallel-sided median lobe of the aedeagus and a strongly sinuate basal margin to the parameres in lateral aspect (Fig. 5).

#### Description.

**Figure 3.**
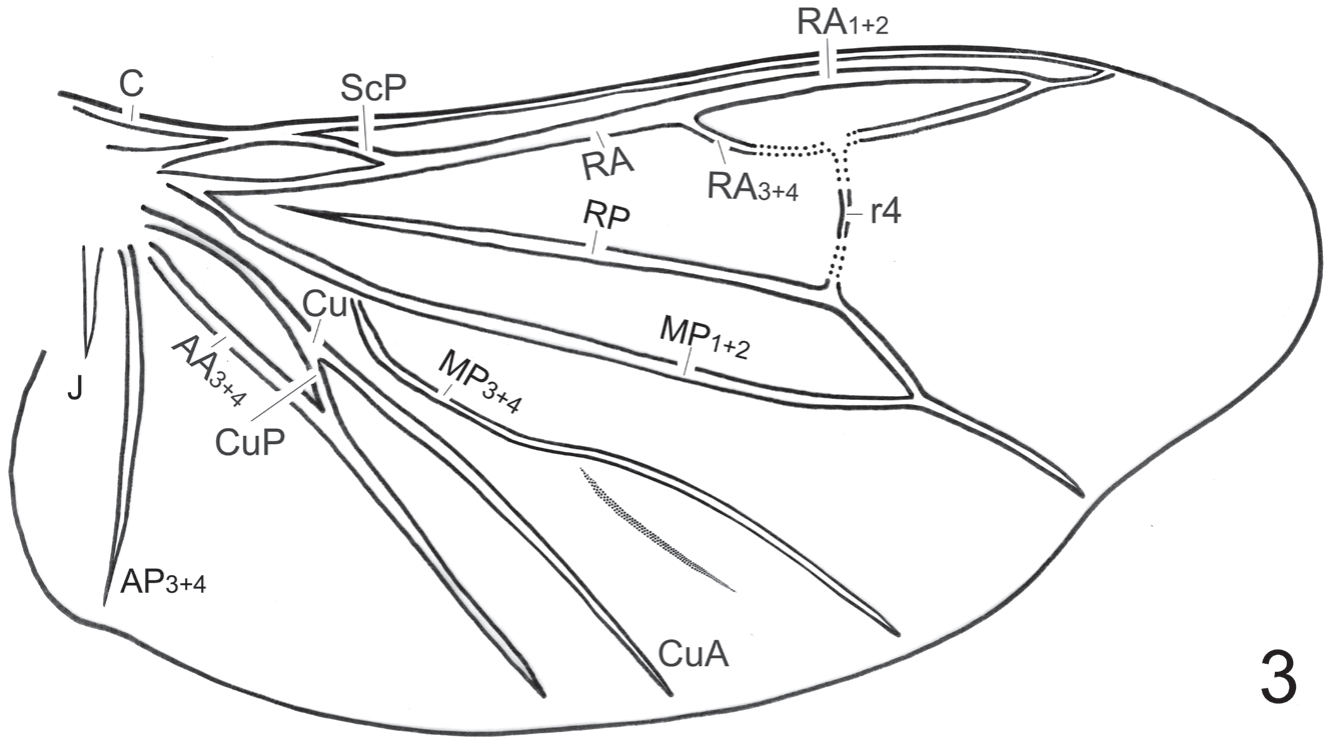
Sketch of right hind wing of *Oculogryphus shuensis* sp. n., male, modified from that of *Oculogryphus bicolor* to show venation.

**Figures 4–5.**
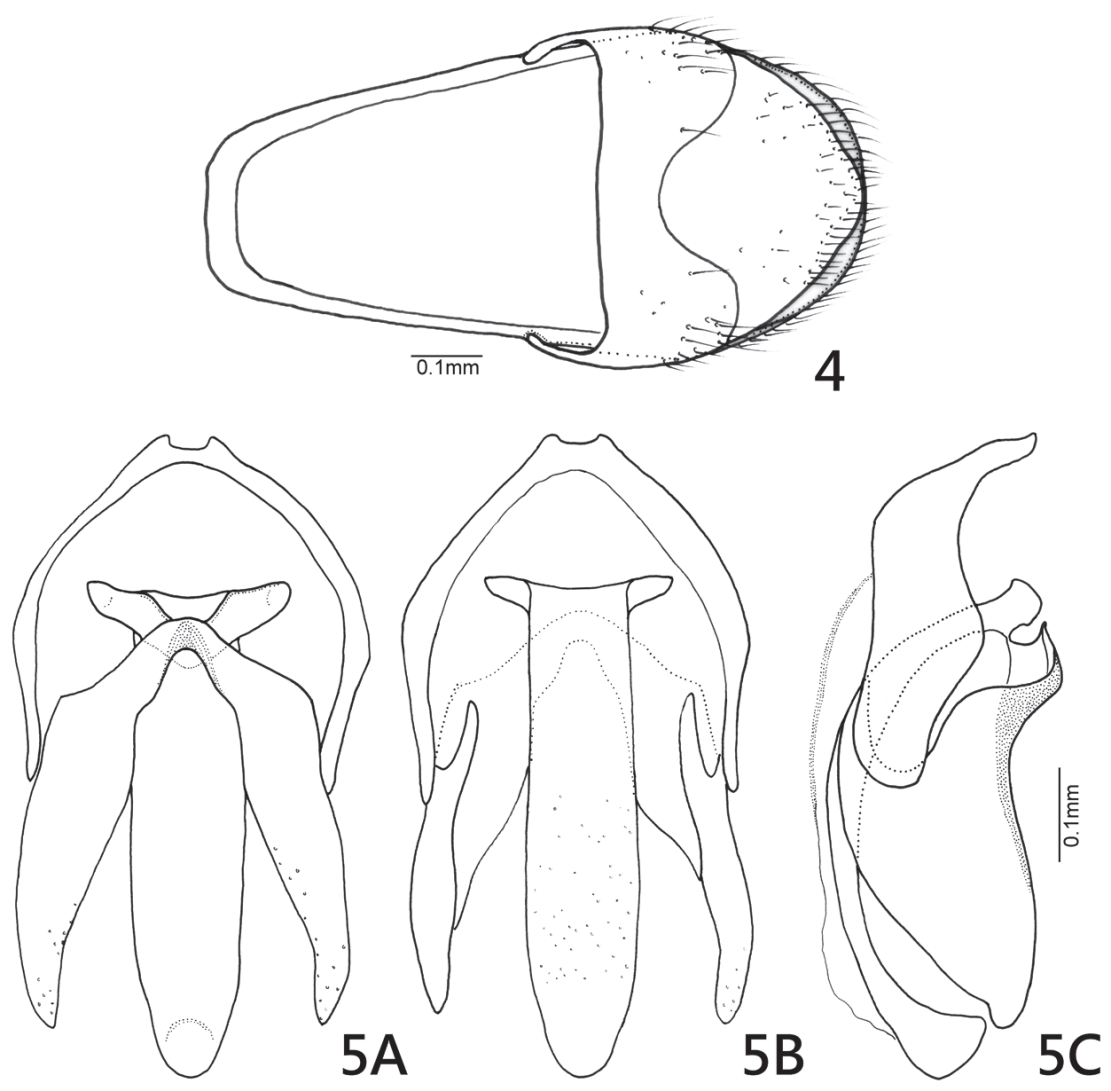
*Oculogryphus shuensis* sp. n., male. **4** aedeagal sheath, dorsal aspect **5** male genitalia, dorsal (**A**), ventral (**B**), and lateral (**C**) aspects.

♂: BL: 6.7–7.1 mm; BW: 2.8–3.1 mm; PW/PL = 1.5–1.6; EL/ EW = 3.4–3.7; EL/PL = 3.7–3.9; BW/PW = 1.3–1.4. The species is very similar to *Oculogryphus bicolor* in general morphology and those characteristics need not be repeated here (*vide* Description of *Oculogryphus bicolor* in Jeng et al. 2011). As described for *Oculogryphus fulvus* and *Oculogryphus bicolor* except: head capsule and antennae black; pronotum and mesoscutellum orange; elytra and epipleura opaquely black except humeri brown; thoracic sternites yellowish brown; all coxae, trochanters and subapices of femora yellowish brown, other parts of legs otherwise black; abdominal ventrites 1–5 and basal half of 6 opaquely black, apical half of 6 and 7–8 yellowish brown. Venation of hind wing (Fig. 3) similar to that of *Oculogryphus fulvus*, with MP_4_ absent or faint. Aedeagal sheath about 0.89 mm in length and 0.42 mm in width; abdominal tergites IX and X clearly recognizable individually; sternite IX with basal corners somewhat squared (Fig. 4). Aedeagus (Fig. 5) about 0.66 mm in length and 0.37 mm broad; median lobe slightly surpassing parameres apically, subparallel-sided dorso-ventrally, with apex significantly dilated in lateral aspect; parameres elongate dorso-ventrally, strongly sinuate on basal margin laterally; basal piece somewhat horseshoe-shaped, with a median notch in caudal margin.

♀: Unknown.

#### Etymology.

The specific epithet is derived from the old name of Sichuan (Shu), where the new species was found.

#### Phenology.

Males appear at least in June.

### Key to species of *Oculogryphus*

**Table:** 

1	Pronotum and elytra highly contrasting in ground coloration, elytra black and pronotum orange; abdominal ventrites 1–5 and basal half of 6 black, apical half of 6 and 7–8 yellowish brown (China: Sichuan)	*Oculogryphus shuensis* sp. n.
–	Pronotum and elytra sharing similar or identical ground coloration, mostly yellowish brown; abdominal ventrites exclusively yellowish brown	2
2	Elytra with base, lateral margins, and sutures yellowish brown and disc smoky brown; body size larger (body length 6.2–8.2 mm); male genitalia with median lobe slightly surpassing apex of parameres (Vietnam)	*Oculogryphus bicolor* Jeng, Engel & Branham
–	Elytra more or less uniformly brown in coloration; body size smaller (body length 6.0 mm); male genitalia with median lobe far surpassing apex of parameres by about 1/3 length of median lobe (Vietnam)	*Oculogryphus fulvus* Jeng

## Discussion

We examined the type material of the type species and several other species of the ototretine genera deposited in the Muséum national d’Histoire naturelle, Paris (MNHN). *Oculogryphus* is doubtless a member of the newly-defined Ototretinae and appears allied to a subgroup whose lateroposterior angles of the pronotum are less prominent. Following Janisova and Bocakova’s (2013) key, *Oculogryphus* falls intermediate between *Falsophaeopterus* Pic and *Stenocladius* in couplet 17 – it shares filiform antennae with *Falsophaeopterus* but has an aedeagal morphology more resembling that of *Stenocladius*. Although similar, the morphological particulars of *Oculogryphus* differ notably from the other two genera. For example, the filiform antennae of *Oculogryphus* are comparatively short and slender in relation to those of *Falsophaeopterus* which are more or less depressed, long and varyingly serrate. As to the aedeagus, the length of the parameres in relation to the phallus (median lobe) and whether the phallobase (basal piece) has a marginal emargination were used to separate *Stenocladius* from *Falsophaeopterus* in Janisova and Bocakova’s (2013) key. It is true that the parameres of *Falsophaeopterus* (including its subgenus *Mimophaeopterus* Pic) are about as long as the phallus, but this is quite variable in *Stenocladius* (as long as 2/3 of the phallus to slightly shorter (6/7), (*cf.* Kawashima 1999, Janisova and Bocakova 2013)). The ratios of phallus/phallobase and paramere/phallobase also varied greatly among species of *Stenocladius* but seem stable in *Falsophaeopterus*, which always has the phallobase shortest among the aedeagal sclerites. The ratios of aedeagal sclerites of *Oculogryphus* show a pattern similar to that observed for *Stenocladius* (Jeng et al. 2007, 2011, present study). The notch on caudal margin of the phallobase is quite clear in the type species of *Stenocladius*, *Stenocladius davidis* Fairmaire (Janisova and Bocakova 2013), but is faint or absent in some others (Kawashima 1999). Similar variation exists among species of *Oculogryphus*, too (Jeng et al. 2007, 2011, present study). The aforementioned features appear not sufficiently reliable to be diagnostically meaningful in differentiating these genera in a key.

*Oculogryphus* is distinct from all of the other ototretine genera by its large compound eyes which are nearly contiguous ventrally and significantly emarginate on the posterior upper margin in males. The genae are mostly vertical, deeply lying between the enlarged compound eyes and separated by a fused gular suture (Fig. 2). Within Ototretinae, only a few unidentified species of *Stenocladius* from China and SE Asia are comparable to *Oculogryphus* in terms of compound eye size. Generally the compound eyes of species of *Stenocladius* are of median size, hemispherical frontally, nearly spherical laterally and separated from each other ventrally by 0.9× to 1.5× ventral width of an individual compound eye (Janisova and Bocakova 2013). The genae and gula between the compound eyes are visible ventrally. In contrast, the large-eyed species of *Stenocladius* have their compound eyes approximate ventrally, separated from each other by scarcely visible genae and a narrow gula and are weakly and broadly-rounded emarginate on the posterior margin. Simultaneously, they also possess shorter antennae and antennal branches in contrast to the typical species of the genus. The combination of enlarged compound eyes and short antennae is likely a character suite adapted to a visually-oriented mate-searching strategy at night. Though enlarged and deeply emarginate compound eyes have evolved in parallel or by convergence among several lampyrid (Ototretinae, Luciolinae, and Lampyrinae) and rhagophthalmid (*Rhagophthalmus* Motschulsky, *Menghuoius* Kawashima and Satô, and *Dioptoma* Pascoe) lineages (Jeng et al. 2007), those of *Oculogryphus* are unique to the newly-defined Ototretinae. Herein we proposed a modification to Janisova and Bocakova’s (2013) key, partially adopted from Jeng et al. (2007) to include *Oculogryphus*:

**Table:** 

17	Tibial spurs present; antennae serrate or filiform, with first flagellomere thicker or broader than scape if filiform	*Falsophaeopterus* Pic
–	Tibial spurs absent; antennae flabellate or filiform, with first flagellomere as thick as or more slender than scape if filiform	18
18	Antennae filiform and short, reaching elytral base at most when in repose; compound eyes large, separated from each other by about half compound eye diameter frontally, ovoid in shape laterally, and with posterior upper margin deeply emarginate in about right angle	*Oculogryphus* Jeng, Engel & Yang
–	Antennae flabellate and long, reaching basal third or half of elytra when in repose; compound eyes medium to large, separated from each other by at least one compound eye diameter frontally, more or less round in shape laterally, with posterior upper margin never deeply emarginate in right angle	*Stenocladius* Fairmaire

## Supplementary Material

XML Treatment for Oculogryphus shuensis
